# Clinical features combined with ultrasound-based radiomics nomogram for discrimination between benign and malignant lesions in ultrasound suspected supraclavicular lymphadenectasis

**DOI:** 10.3389/fonc.2023.1048205

**Published:** 2023-03-09

**Authors:** Jieli Luo, Peile Jin, Jifan Chen, Yajun Chen, Fuqiang Qiu, Tingting Wang, Ying Zhang, Huili Pan, Yurong Hong, Pintong Huang

**Affiliations:** ^1^ Department of Ultrasound in Medicine, Zhejiang University School of Medicine Second Affiliated Hospital, Zhejiang University, Hangzhou, China; ^2^ Research Center of Ultrasound in Medicine and Biomedical Engineering, Zhejiang University School of Medicine Second Affiliated Hospital, Zhejiang University, Hangzhou, China; ^3^ Research Center for Life Science and Human Health, Binjiang Institute of Zhejiang University, Hangzhou, China

**Keywords:** supraclavicular lymph node, radiomics, ultrasound, nomogram, lymphadenectasis

## Abstract

**Background:**

Conventional ultrasound (CUS) is the first choice for discrimination benign and malignant lymphadenectasis in supraclavicular lymph nodes (SCLNs), which is important for the further treatment. Radiomics provide more comprehensive and richer information than radiographic images, which are imperceptible to human eyes.

**Objective:**

This study aimed to explore the clinical value of CUS-based radiomics analysis in preoperative differentiation of malignant from benign lymphadenectasis in CUS suspected SCLNs.

**Methods:**

The characteristics of CUS images of 189 SCLNs were retrospectively analyzed, including 139 pathologically confirmed benign SCLNs and 50 malignant SCLNs. The data were randomly divided (7:3) into a training set (n=131) and a validation set (n=58). A total of 744 radiomics features were extracted from CUS images, radiomics score (Rad-score) built were using least absolute shrinkage and selection operator (LASSO) logistic regression. Rad-score model, CUS model, radiomics-CUS (Rad-score + CUS) model, clinic-radiomics (Clin + Rad-score) model, and combined CUS-clinic-radiomics (Clin + CUS + Rad-score) model were built using logistic regression. Diagnostic accuracy was assessed by receiver operating characteristic (ROC) curve analysis.

**Results:**

A total of 20 radiomics features were selected from 744 radiomics features and calculated to construct Rad-score. The AUCs of Rad-score model, CUS model, Clin + Rad-score model, Rad-score + CUS model, and Clin + CUS + Rad-score model were 0.80, 0.72, 0.85, 0.83, 0.86 in the training set and 0.77, 0.80, 0.82, 0.81, 0.85 in the validation set. There was no statistical significance among the AUC of all models in the training and validation set. The calibration curve also indicated the good predictive performance of the proposed nomogram.

**Conclusions:**

The Rad-score model, derived from supraclavicular ultrasound images, showed good predictive effect in differentiating benign from malignant lesions in patients with suspected supraclavicular lymphadenectasis.

## 1 Introduction

Supraclavicular lymphadenectasis has been frequently observed in patients with benign diseases such as reactive hyperplasia, tuberculosis, granulomatous inflammation, etc., and malignant diseases such as lung cancer metastasis, breast cancer metastasis, esophageal cancer metastasis, etc (1). Thus, it is important to distinguish between benign and malignant supraclavicular lymph nodes (SCLNs) for the further treatment of patients (2, 3). Computerized tomography (CT) examination is based on the density of lymph nodes and surrounding soft tissue to distinguish, the enlarged supraclavicular lymph nodes are often indistinguishable from the surrounding tissues and muscles on plain CT scan (4). Due to the fixed and superficial location of the supraclavicular region and clear supraclavicular anatomical structure, high frequency conventional ultrasound (CUS) has its unique advantages for SCLNs examination. CUS is preferred as the first choice for SCLNs with high resolution, low cost and no radiation. However, there is overlap between benign and malignant images for atypical CUS features.

Radiomics, a process of converting radiographic images into quantifiable information, can provide more comprehensive and richer information than radiographic images, which are imperceptible to human eyes (5, 6). Imaging examination is one of the routine steps in routine clinical diagnosis, so radiomics research based on images has certain feasibility. Some studies showed that radiomics analysis on CUS images can effectively predict malignant parotid gland lesions (7). Some studies have shown that image feature-based radiomics extraction has objective characteristics and great value in predicting central lymph node metastasis in papillary thyroid carcinoma patients with Hashimoto’s thyroiditis (8). However, the clinical value of CUS-based radiomics analysis to differentiate of benign and malignant SCLNs was unknown.

Therefore, the purpose of this investigation was to extract the radiomics parameters from supraclavicular CUS images and to establish predictive a nomogram radiomics score (Rad-score) model to noninvasively identify benign and malignant SCLNs.

## 2 Materials and methods

### 2.1 Patients

Between January 2021 and July 2022, a total of 189 patients participated in the retrospective study, including pathologically confirmed 50 benign lesions and 139 malignant lesions. The inclusion criteria were as follows: (1) patients with CUS suspected SCLNs; (2) patients who underwent pathological examination within 2 weeks after a SCLNs CUS examination; (3) patients who had high-quality ultrasound image; (4) patients who had definite pathological findings. The exclusion criteria were as follows: (1) patients who underwent previous SCLN treatment (resection biopsy, radiotherapy, chemotherapy); (2) patients who had poor ultrasound image quality; (3) patients who had incomplete clinical data. (4) patients who missed histopathological results. (5) lesion larger than 5 cm in diameter due to the limited width of the US probe.

### 2.2 Ultrasound examination and pathological examination

The CUS examination was performed using GE LOGIQ E9, Esaote Mylab 90, Toshiba Aplio 500, Mindray Resona 7, and Philips iU22 with corresponding high-frequency linear array probes. Each patient was placed in the supine position while lying on the examination bed. Then, the neck was fully extended, and the patient was told to breathe calmly. The SCLNs were examined by 3-year experience radiologist in superficial CUS. If there were multiple lesions, then the most suspicious would be first recommended for further pathological examination. If the most suspicious one is not suitable for biopsy, the largest one (short diameter) would be second recommended for further pathological examination. The boundary (clear and unclear), shape (long/short diameter < 2, long/short diameter ≥ 2), calcification (with calcification, without calcification), hilus (present or absent), margin (well-defined and ill-defined), structure (cystic or hyperechoic nodule, no cystic and hyperechoic nodule) of the SCLNs were observed and recorded. The most suspected supraclavicular lymphadenectasis were included in the study. CUS suspected SCLN was submitted to ultrasound guided biopsy at ultrasound department. The pathological section was read by a pathologist with more than 5 years of experience. For the pathological benign SCLN, we followed up at least for 6 months. No benign SCLN had progression on ultrasound image.

### 2.3 Region of interest (ROI) segmentation and feature extraction

Firstly, the patients were randomly divided into the training and validation groups in a 7:3 ratio. The jpg format ultrasound images were exported from the imaging system and imported into PyCharm software (Community edition 2022.2). All images were resampled and normalized before feature extraction (7). Then, the ROI was manually segmented by an experienced radiologist and confirmed by another one by using Labelme (3.16.7) software package. Both were blinded to the pathological results before performing image annotation, and consensus was reached by discussion in cases of disagreement. Finally, the radiomics data was extracted by python package pyradiomics (V3.0.1) (https://pyradiomics.readthedocs.io/en/latest/features.html) and 744 radiomics features from SCLNs were extracted in this study.

### 2.4 Radiomics feature selection

Radiomics features were extracted from lesions after image processing with different filters by using the open-source Pyradiomics package V3.0.1 and were divided into the following classes: (a) first-order statistics; (b) shape-based features; (c) high-order features, including gray-level co-occurrence matrix (GLCM), gray-level size zone matrix (GLSZM), gray-level run length matrix (GLRLM), gray-level dependence matrix (GLDM), and neighboring gray tone difference matrix (NGTDM). Firstly, independent *t*-test was used to select significant features with statistically significant difference (P < 0.05). Then, the least absolute shrinkage and selection operator (LASSO) was used to select nonzero coefficients by 10-fold cross validation. Finally, Rad-score was calculated based on the selected features.

### 2.5 Models construction

The clinical and CUS data were analyzed by multivariate analysis firstly to select the statistically significant predictors of distinguishing between benign and malignant lesions and follow by logistic regression analysis. The Rad-score model, CUS model, radiomics-CUS (Rad-score + CUS) model, clinic-radiomics (Clin + Rad-score) model, and combined CUS-clinic-radiomics (Clin + CUS + Rad-score) model were established both in training and validation sets. The calibration curve was used to evaluate the calibration ability. The prediction accuracy of the models was represented by the receiver operating characteristic (ROC) curve and was quantified by the area under the ROC curve (AUC) in both the training and test sets.

### 2.6 Statistical analysis

Statistical analyzes were performed using statistical software for Windows version 23.0 (SPSS Inc., Chicago, IL, USA) and R software version 4.2.1 (R project for statistical computing). Quantitative data with abnormal distribution was expressed as median (interquartile range 25^th^, 75^th^ percentile). Quantitative data with normal distribution was expressed as mean ± standard deviation. Wilcox test was conducted to compare the data displaying an abnormal distribution. Statistical analysis was by chi-squared test when comparing categorical variables. The LASSO method constructed a penalty function by adding constraint conditions, and a prediction model was constructed by performing a 10-fold cross-validation. The models were built using logistic regression and the diagnostic performance for differentiating between benign and malignant lesions was using ROC. DeLong’s test was used to evaluate different ROC curves. Calibration curves were constructed to assess the predictive value of different models. A *P* value of less than 0.05 was considered statistically significant.

## 3 Results

### 3.1 Basic information of patients with SCLNs lymphadenectasis

**Figure 1:**
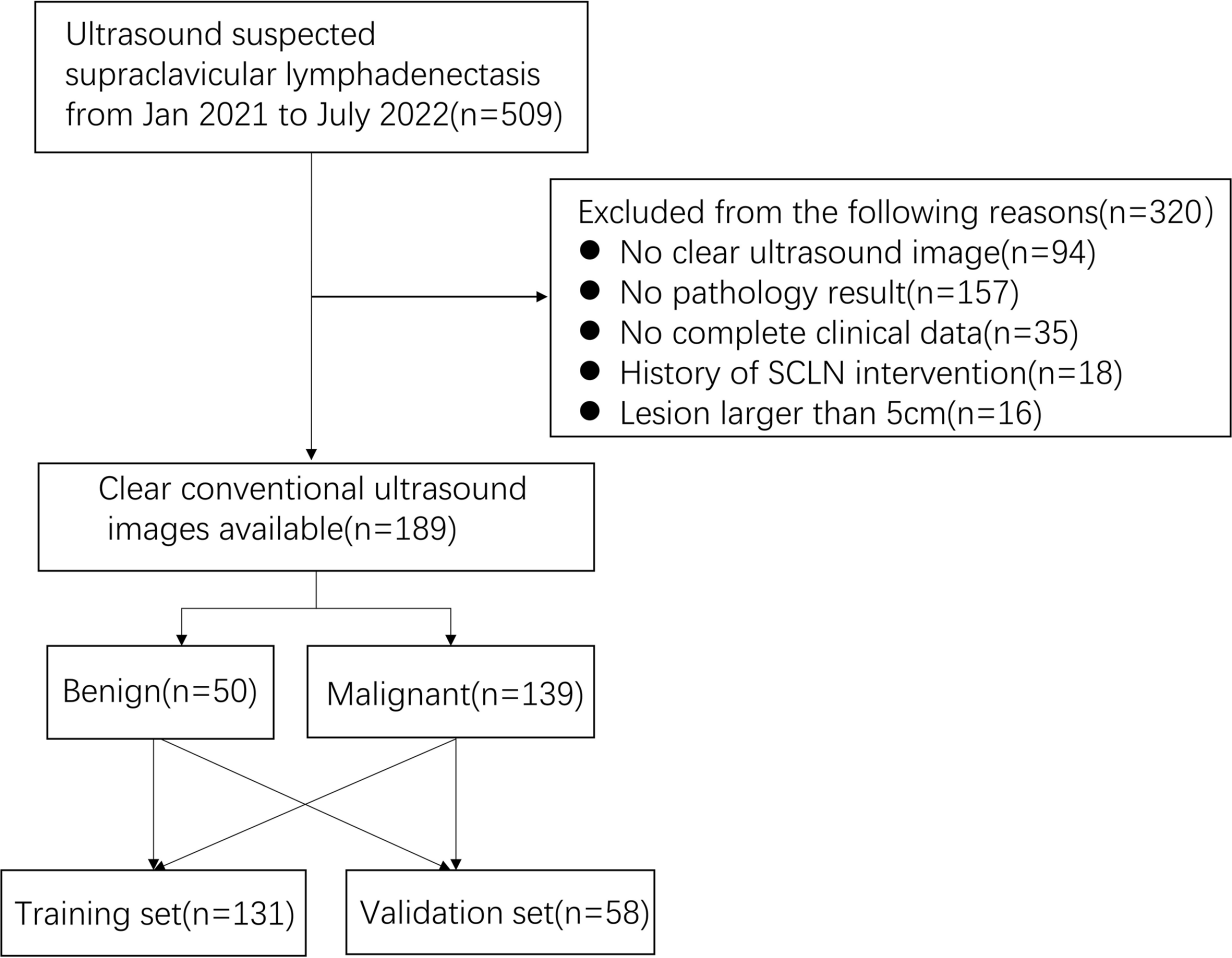
The flowchart of patient selection for dividing into the training set and validation set.

**Figure 2:**
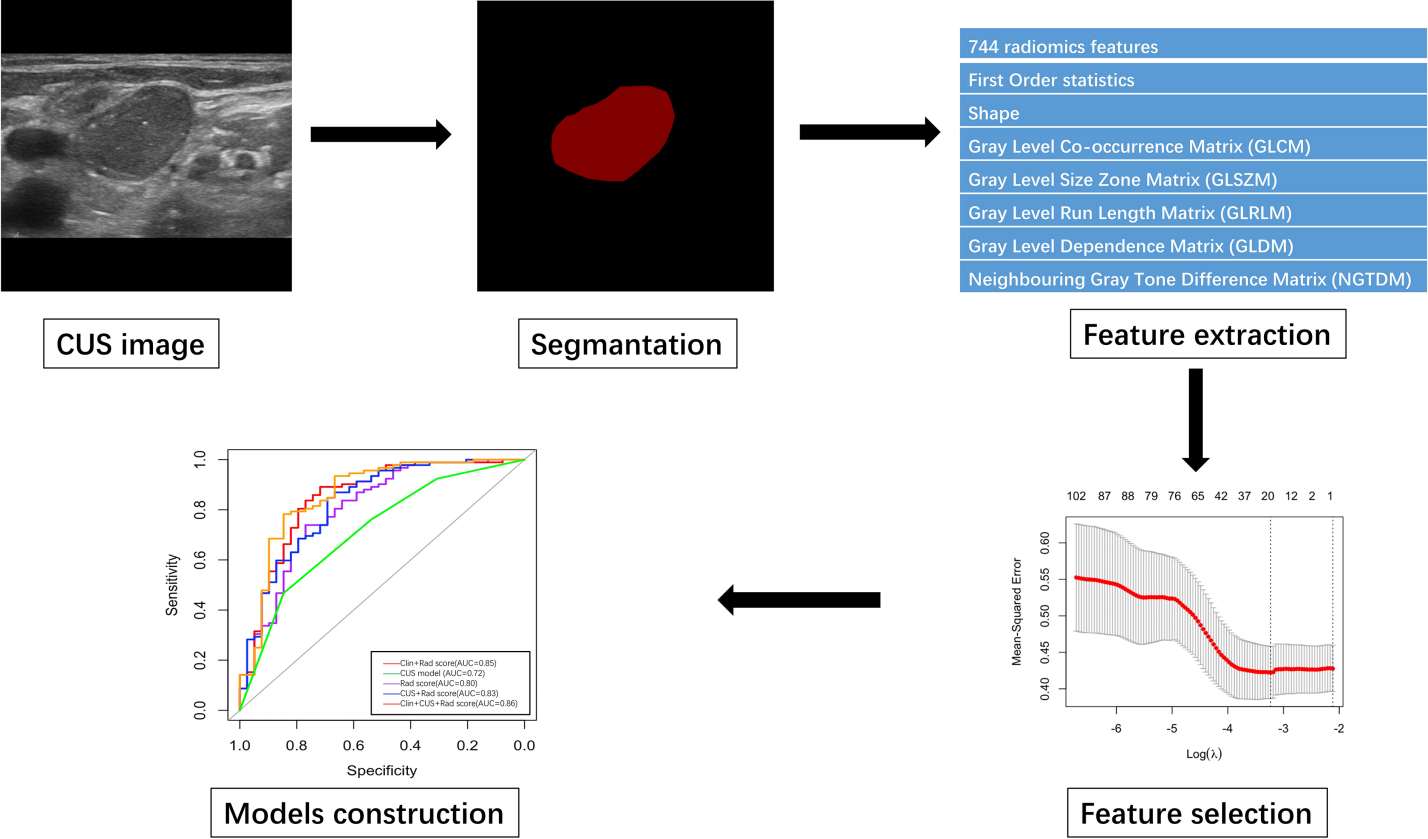
The flowchart of CUS segmentation, feature extraction, feature selection and models construction.

A total of 189 patients were recruited (**Figures 1**, **2**), including 50 pathologically confirmed benign (16 males and 34 females; age 59.5 (44.5-67.75) years) and 139 malignant (73 males and 66 females; age 65 (53.5-70) years). The specific pathological findings of 50 benign and 139 malignant SCLNs lymphadenectasis was showing (**Supplemental Table 1**). The median follow-up after biopsy for benign SCLNs lymphadenectasis was 10 (range, 6-20) months. No benign SCLN had progression on ultrasound image. The SCLNs malignant rates of the training set and the validation set were 70.23% and 81.03%, respectively. There were no significant differences in patient age, sex, location, pathology, diameter, tumor history, boundary, margin, calcification, shape, hilus, structure, and rad-score between the training set and the validation set (*P*>0.05), as shown in **Table 1**.

**Table 1:** Clinic-CUS characteristics of suspected supraclavicular lymphadenectasis between training and validation set.

Characteristic	Training set	Validation set	*P* value
Age(year)	65 (51,70)	62 (52.25,68.75)	0.73
Diameter (cm)	2.11 (1.28,2.77)	2.19 (1.7,2.9)	
Sex (%)			0.80
Male	63 (48%)	26 (45%)	
Female	68 (52%)	32 (55%)	
Location (%)			0.06
Left	74 (56%)	42 (72%)	
Right	57 (44%)	16 (28%)	
Tumor history			0.56
No	82 (63%)	33 (57%)	
Yes	49 (37%)	25 (43%)	
Pathology			0.17
Benign	39 (30%)	11 (19%)	
Malignant	92 (70%)	47 (81%)	
Boundary			0.12
Clear	85 (65%)	37 (64%)	
Unclear	46 (35%)	21 (36%)	
Margin			0.19
Well-defined	58 (44%)	19 (33%)	
Ill-defined	73 (56%)	39 (67%)	
Calcification			0.99
No calcification	97 (74%)	43 (74%)	
Calcification	34 (26%)	15 (26%)	
Shape			0.61
Long/short diameter≥2	43 (33%)	22 (38%)	
Long/short diameter<2	88 (67%)	36 (62%)	
Hilus			0.71
Present	14 (11%)	8 (14%)	
Absent	117 (89%)	50 (86%)	
Structure			0.11
No cystic and hyperechoic nodule	125 (95%)	51 (88%)	
Cystic or hyperechoic nodule	6 (5%)	7 (12%)	
Rad-score	0.98 (0.71,1.19)	0.95 (0.77,1.15)	0.86

CUS, conventional ultrasound.

### 3.2 Univariate analysis of clinical features, CUS features, and Rad-score for malignant lesions

Benign lesions were more likely to be found with no tumor history (*P*<0.05, **Table 2**) in the training set. The validation set also found the same findings. For CUS features, well-defined margin, clear boundary, and long/short diameter≥2 shape were commonly observed in benign lesions (all *P*<0.05) in the training set. The same results were also found in the validation set.

**Table 2:** Univariate analysis of clinical information, CUS features, and Rad-score for distinguishing benign from malignant lesions in the training set.

	Benign	Malignant	*P* value
Clinical information			
Age (year)	57 (45, 67.5)	65.5 (54.75, 70)	0.093
Male/Female	13/26	50/42	0.044
Diameter (cm)	2.11 (1.28, 2.77)	2.1 (1.35, 3.04)	0.678
Tumor history (N/Y)	33/6	49/43	0.001
CUS feature			
Boundary (clear/unclear)	31/8	54/38	0.038
Margin (well-defined/ill-defined)	24/15	34/58	0.016
Calcification (no calcification/calcification)	27/12	70/22	0.548
Shape (long/short diameter ≥ 2/< 2)	21/18	22/70	0.002
Hilus (present/absent)	4/35	10/82	1.000
Structure (no cystic and hyperechoic nodule/cystic or hyperechoic nodule)	38/1	87/5	0.669
CUS-radiomics			
Rad-score	0.64 (0.16,0.9)	1.08 (0.87,1.26)	<0.001

CUS, conventional ultrasound.

**Figure 3:**
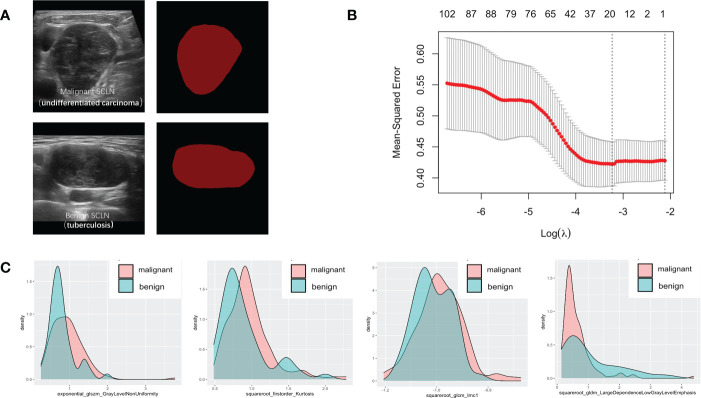
Variables extracted from benign and malignant supraclavicular lymph node. **(A)** The ROI of benign and malignant supraclavicular lymph node. **(B)** The Mean-square error plot of LASSO regression in supraclavicular lymph node. **(C)** The density plots between extracted radiomics variables in benign and malignant supraclavicular lymph node.

Seven hundred and forty-four radiomic features were extracted from each ROI, and a total of 20 radiomic features (**Supplemental Table 2**) with non-zero coefficients were screened out based on t test and LASSO (**Figure 3**). The Rad-score was calculated between benign and malignant lesions (**Supplemental Table 3**). The results showed that patients with benign lesions had lower Rad-score than patients with malignant lesions, which was statistically significant in both training (0.64(0.16, 0.9) vs. 1.08(0.87, 1.26), *P* < 0.05) and validation sets (0.63(0.13, 0.89) vs. 0.98(0.81, 1.24), *P* < 0.05).

### 3.3 Multivariate analysis of variables for predicting malignant lesions

In the multivariate analysis of clinical information, CUS features and Rad-score characteristics, tumor history (OR=4.88, 95%CI 1.65-17.24), long/short diameter < 2 shape (OR=2.69, 95%CI 1.04-7.14), Rad-score (OR=15.57, 95%CI 5.20-64.49) were significantly correlated with pathological results (*P*<0.05). However, sex, boundary, and the margin were not independent signatures for predicting malignant lesions (**Supplemental Table 4**).

### 3.4 Performance, construction and validation of nomogram

**Figure 4:**
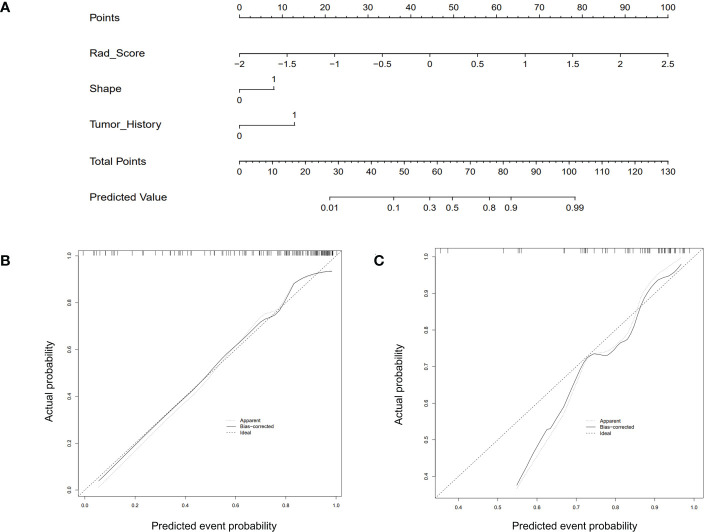
The CUS-based radiomics nomogram and calibration curves of the nomogram. **(A)** Integrating Rad-score, shape, and tumor history, the CUS-based nomogram was established. Calibration curves of the nomogram in the training **(B)** and testing **(C)** set.

**Figure 5:**
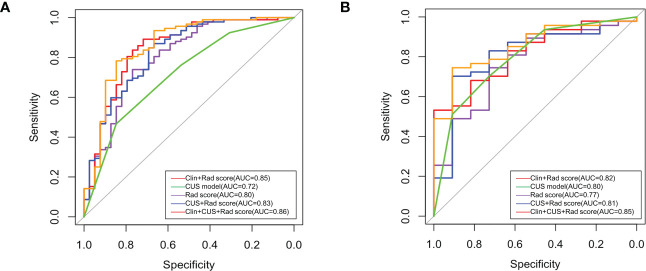
Receiver operating characteristic (ROC) curves of all models in training set **(A)** and validation set **(B)**.

The Rad-score model, Clin + Rad-score model, Rad-score + CUS model, and Clin + CUS + Rad-score model were constructed based on clinical information, CUS features and Rad-score (**Figure 4**). The AUC of CUS model, Rad-score model, Clin + Rad-score model, Rad-score + CUS model, and Clin + CUS + Rad-score model were 0.72, 0.80, 0.85, 0.83 and 0.86 in the training set, respectively (**Figure 5**; **Table 3**). The AUC of CUS model, Rad-score model, Clin + Rad-score model, Rad-score + CUS model, and Clin + CUS + Rad-score model were 0.80, 0.77, 0.82, 0.81 and 0.85 in the validation set. By the Delong test, there was no significant difference in the AUC of the Rad-score between the training set and the validation set (*p*=0.70). There were no significant differences among all models in terms of AUC. A nomogram that contains Rad-score, shape and tumor history variables describing SCLNs to predict the malignant lesion with CUS suspected supraclavicular lymphadenectasis.

**Table 3:** The ROC, accuracy, sensitivity, and specificity of five models in the training set and validation set.

Group	Model	AUC (95%CI)	Accuracy	Sensitivity	Specificity
Training set	Rad-score	0.80 (0.71-0.89)	0.75	0.74	0.77
	CUS	0.72 (0.62-0.81)	0.58	0.47	0.85
	Clin + Rad-score	0.85(0.77-0.93)	0.84	0.89	0.72
	CUS + Rad-score	0.83(0.75-0.91)	0.80	0.85	0.69
	Clin + CUS + Rad-score	0.86(0.78-0.94)	0.80	0.78	0.85
Validation set	Rad-score	0.77(0.61-0.92)	0.74	0.75	0.73
	CUS	0.80 (0.65-0.94)	0.71	0.70	0.73
	Clin + Rad-score	0.82(0.69-0.94)	0.62	0.53	1.00
	CUS + Rad-score	0.81(0.66-0.96)	0.74	0.70	0.91
	Clin + CUS + Rad-score	0.85(0.74-0.96)	0.78	0.75	0.91

AUC, area under the curve; CI, confidence interval; Rad-score, radiomics score; Clin, Clinical; CUS, common ultrasound.

## 4 Discussion

SCLN metastasis is of great significance for treatment decision-making and prognostic evaluation (9–11). Among all imaging methods, CUS is considered the most convenient method for assessing the characteristics of SCLN. However, the diagnostic value of CUS in assessing lymph nodes is controversial. Zheng et al.’s study reported that the diagnostic performance of axillary CUS was poor with an AUC of 0.585-0.719 (12). In our study, several clinical and ultrasound imaging features were associated with the differential diagnosis of benign and malignant lesions. Benign SCLNs were more likely to have no tumor history, which was consistent with previous research (13). For CUS features, well-defined margin, clear boundary, and long/short diameter ≥ 2 shape were commonly observed in benign lesions, but also occasionally seen in malignant lesions (14). The overlapping of CUS features and clinic data brings some difficulties in the diagnosis of benign and malignant lymph nodes.

CUS-based radiomics could provide a large number of quantitative image features from ultrasonic images, which tend to be hard for the naked eyes to recognize (7). Seven hundred and forty-four radiomics features were selected from SCLN. These included first-order statistics and high-order features with various filters (NGTDM, GLRLM, GLSZM, GLCM, and GLDM). First-order statistics, also known as grayscale histogram features, are mainly used to perform statistical calculations on the entire image or the ROI within the image, and are used to describe the grayscale of the image. Second-order statistics refer to the spatial relationship between the intensities of each voxel. Higher-order statistics are used for feature extraction and image preprocessing such as wavelet decomposition, Fourier transform and other filtering (15). The software automatically extracts radiomics features to compensate for errors introduced by manual and subjective measurements. In our results, the AUCs of Rad-score model, Clin + Rad-score model, Rad-score + CUS model, and Clin + CUS + Rad-score model were 0.80, 0.85, 0.83, 0.86 in the training set and 0.77, 0.82, 0.81, 0.85 in the validation set. There were no significant differences among all models in terms of AUC. Zhou et al. (16) developed an ultrasound radiomics nomogram to identify central lymph node metastasis in patient papillary thyroid cancer, the AUCs for the training set, internal validation set, and external validation set were 0.816 and 0.858, respectively. Radiomics based on analysis of CUS images showed good performances as other routine methods. Through the Rad-score model, we could distinguish benign and malignant lesions when supraclavicular lymphadenopathy is suspected on ultrasonography.

This study has some limitations. First, the overall sample size is small. The sample size should be expanded in future studies. Second, this was a single-center retrospective study with good predictive power, which may indicate the need for an external validation set to validate this predictive model. Third, the study included several different ultrasound machines that could have affected the findings. Fourth, the biopsy specimen had a possibility of false negative. In the future, large, multi-center clinical studies, more subgroups and enhanced ultrasound images are needed to further confirm the findings of this study.

## 5 Conclusion

In conclusion, the radiomics nomogram, derived from CUS, showed favorable prediction efficacy for differentiating benign from malignant in patients with suspected supraclavicular lymphadenectasis. The Rad-score model improves the differentiation of the benign lesion from malignant lesion.

## Data availability statement

The raw data supporting the conclusions of this article will be made available by the authors, without undue reservation.

## Ethics statement

The retrospective study was approved by the ethics consultant committee of Zhejiang University School of Medicine Second Affiliated Hospital. Written informed consent for participation was not required for this study in accordance with the national legislation and the institutional requirements.

## Author contributions

JL and PJ contributed equally to this study. JL and PJ were responsible for the conception and design of this study. JL and JC contributed to the data analysis and writing of the manuscript. YC, FQ, and TW contributed to data collection. YH, YZ, HP, and PJ contributed to data analysis and manuscript preparation. PH contributed to writing-reviewing. All authors contributed to the article and approved the submitted version.
