# Supplementary material for: Clinical features combined with ultrasound-based radiomics nomogram for discrimination between benign and malignant lesions in ultrasound suspected supraclavicular lymphadenectasis
Source: Front Oncol. 2023 Mar 9;13:1048205. doi: 10.3389/fonc.2023.1048205 (PMC10034097; doi:10.3389/fonc.2023.1048205)
Supplement: Supplementary file 1 [file Table_1.docx]

**Supplemental Information**

**Supplemental Table 1. The specific pathological findings of 50 benign and 139 malignant SCLNs lymphadenectasis.**

|  | **Number** |
| --- | --- |
| **Benign** |  |
| Granulomatous inflammation | 14 |
| Reactive lymphoid hyperplasia | 20 |
| Proliferating lymphoid tissue | 6 |
| Chronic lymphadenitis | 4 |
| Tuberculosis | 3 |
| Necrotic tissue | 3 |
| **Malignant** |  |
| Metastatic lung cancer | 45 |
| Metastatic breast cancer | 16 |
| Metastatic cervical cancer | 2 |
| Metastatic gastric cancer | 2 |
| Metastatic prostate cancer | 3 |
| Metastatic endometrial cancer | 1 |
| Metastatic bowel cancer | 5 |
| Metastatic urothelial cancer | 1 |
| Metastatic esophageal cancer | 2 |
| Metastatic mediastinal cancer | 1 |
| Metastatic cancer (no clear indication) | 32 |
| Infiltration of cancer cells (no specific cancer) | 16 |
| Plasmacytoma | 1 |
| Lymphoma | 12 |

**Supplemental Table 2.** The class of extracted variables.

| **Variables Class** | **Number** |
| --- | --- |
| GLCM | 7 |
| GLDM | 1 |
| GLRLM | 4 |
| GLSZM | 3 |
| NGTDM | 3 |
| First order | 2 |

GLCM, Gray Level Co-occurrence Matrix; GLDM, Gray Level Dependence Matrix; GLRLM, Gray Level Run Length Matrix; GLSZM, Gray Level Size Zone Matrix; NGTDM, Neighbouring Gray Tone Difference.

**Supplemental Table 3. The results of the 20 radiomics features between benign and malignant lesions.**

| **Variables** | **Benign** | **Malignant** |
| --- | --- | --- |
| Exponential_glcm_Idmn | 1 (1, 1) | 1 (1, 1) |
| Exponential_glrlm_GrayLevelNonUniformity | 0.92 (0.63, 1.19) | 0.73 (0.57, 0.88) |
| Exponential_glrlm_GrayLevelVariance | 0.16 (0.04, 0.64) | 0.18 (0.04, 0.61) |
| Exponential_glszm_GrayLevelNonUniformity | 0.92 (0.63, 1.19) | 0.73 (0.57, 0.88) |
| Exponential_glszm_GrayLevelVariance | 0.16 (0.04, 0.64) | 0.18 (0.04, 0.61) |
| Gradient_glcm_Correlation | 1 (0.9, 1.08) | 1.02 (0.95, 1.09) |
| Gradient_glcm_Imc2 | 0.88 ± 0.44 | 0.95 ± 0.4 |
| Gradient_glrlm_GrayLevelNonUniformityNormalized | 0.96 (0.86, 1.15) | 0.93 (0.79, 1.07) |
| Gradient_glrlm_RunVariance | 0.56 (0.36, 1.08) | 0.79 (0.26, 1.32) |
| Gradient_glszm_ZoneVariance | 0.56 (0.36, 1.08) | 0.79 (0.26, 1.32) |
| Gradient_ngtdm_Coarseness | 0.23 (0.08, 0.44) | 0.15 (0.07, 0.3) |
| Logarithm_glcm_ClusterShade | -0.02 (-0.2, 0.01) | -0.01 (-0.15, 0.01) |
| Logarithm_glcm_Imc1 | -0.99(-1.04,-0.93) | -0.99 (-1.06, -0.96) |
| Logarithm_ngtdm_Strength | 0.45 (0.23, 0.87) | 0.57 (0.3, 0.93) |
| Square_glcm_MCC | 1.01 (0.97, 1.04) | 1.01 (0.96, 1.04) |
| Square_ngtdm_Coarseness | 0.03 (0.01, 0.06) | 0.03 (0.02, 0.06) |
| Squareroot_firstorder_Kurtosis | 0.92 (0.78, 1.07) | 0.79 (0.69, 0.99) |
| Squareroot_glcm_Imc1 | -0.99 ± 0.09 | -1.01 ± 0.07 |
| Squareroot_gldm_LargeDependenceLowGrayLevelEmphasis | 0.48 (0.36, 0.76) | 0.69 (0.43, 1.55) |
| Wavelet.H_firstorder_Skewness | -0.01 (-0.03, 0) | 0 (-0.03, 0.05) |

**Supplemental Table 4.** Multivariate analysis of variables for **predicting malignant lesions.**

| **Variabe** | **Odds Ratio (95%CI)** | ***P* value** |
| --- | --- | --- |
| Sex(male) | 2.20 (0.81-6.29) | 0.13 |
| Unclear boundary | 0.89 (0.22-3.51) | 0.86 |
| Ill-defined margin | 1.21 (0.35-4.31) | 0.76 |
| Shape (long/short diameter < 2) | 3.01 (1.12-8.37) | 0.03 |
| Tumor history | 5.16 (1.68-18.89) | 0.007 |
| Rad-score | 13.76 (4.38-58.77) | <0.001 |

CI: confidence interval.
